# Botulinum Toxin—A Current Place in the Treatment of Chronic Migraine and Other Primary Headaches

**DOI:** 10.3390/toxins14090619

**Published:** 2022-09-05

**Authors:** Katarzyna Kępczyńska, Izabela Domitrz

**Affiliations:** Department of Neurology, Medical University of Warsaw, 80 Cegłowska St., 01-809 Warsaw, Poland

**Keywords:** chronic migraine, tension-type headache, cluster headache, onabotulinumtoxinA, headaches, botulinum toxin

## Abstract

Headaches are a very common condition that most people will experience many times during their lives. This article presents the primary headaches, which are a large group of diseases where the headache is not a symptom of another known disease. Tension-type headache affects approximately 80% of the general population, and the prevalence of migraine is estimated at 10–12%. Clinical data and experience to date have demonstrated that botulinum toxin may be an effective prophylactic treatment for chronic headache types. It has been used in neurology for the treatment of dystonia and blepharospasm. Now it has been approved to treat chronic migraine and has been shown to confer significant benefit in refractory cases. Based on clinical experience botulinum toxin has also been tried in other headache disorders. While it is intuitively attractive to think that due to its effect on pain by sensory modulation, there may also be efficacy in its use in chronic tension-type headache and cluster headache, so far, there is little evidence to support this. Botulinum toxin is effective in pain control through its interaction with the SNARE complex, which inhibits the release of neurotransmitters, such as glutamate, substance P and calcitonin gene-related peptide. OnabotulinumtoxinA is effective not only in headache frequency and pain intensity but in other parameters, including quality of life.

## 1. Introduction

Headaches are a very common condition that most people will experience many times during their lives. The World Health Organization (WHO) estimated that almost half of all adults will have experienced at least one headache within the last year. There are several kinds of headaches caused by various factors such as our environment, the medication we take and other causes. The International Classification of Headache Disorders 3rd edition (ICHD-3) defines more than 150 different types of headaches, which it divides into two main categories: primary and secondary [1]. The primary headaches, otherwise named idiopathic, are a large group of diseases and syndromes in which the etiology is unclear, and the headache is not a symptom of another known disease. The most common of these are migraines, tension-type headaches and trigeminal autonomic cephalalgias. On the other hand, secondary headaches may be a symptom of a serious underlying medical condition [2].

Migraines are highly prevalent. As a chronic disease, it is the third most common and the seventh most disabling illness globally in people under 50 years old [3]. Only correct diagnosis and effective treatment positively affect a patient’s quality of life. Chronic migraine (CM) is defined by the current ICHD- 3 as a headache occurring on ≥15 days per month for 3 months with features of migraine on ≥8 days/month and is a disabling condition that effects 0.5% to 5% of the general population [1]. The progression of episodic migraine to chronic migraine is a complex mechanism that is not fully understood. However, known modifiable risk factors for the progression include the frequency of headache attacks, stressful life events and ineffective acute treatment [4].

There are many treatment options available to help manage the pain. Pharmacologic management for primary headaches includes both: acute and prophylactic treatment strategies. However, we often observe considerable side effects with these therapies, which, unfortunately, limits their usefulness. In patients who take medications too often to treat their headaches, MOH—Medication Overuse Headache—may occur. It is also known as a rebound headache. These can cause migraine episodes to occur more frequently and become more severe. Instead of alleviating symptoms, the medications increase the intensity and frequency of headaches [5]. Migraines, if it is not effectively managed, can lead to significant disability. The primary goals of migraine treatment include relieving the pain, reducing headache frequency and preventing progression to chronic migraine.

Migraines are often refractory to medical therapy and may respond well to onabotulinum toxin (ONABoNTA). ONA-BoNTA is used in neurology for the treatment of dystonia and blepharospasm. Now ONA-BoNTA injections are approved for preventing chronic migraines. The clinical efficacy of botulinum toxin serotype A has been shown in two phase III, placebo-controlled trials (PREEMPT 1 and PREEMPT 2) [6,7]. Based on the phase III program, the number of headache days per month was significantly lower. Significant reductions in the frequency of headache days, headache episodes and triptane use were observed. In summary, the authors noted that OnabotulinumtoxinA positively influenced quality of life and had an acceptable safety profile.

## 2. Clinical History of Botulinum Toxin

BoNT is a neurotoxin produced by the bacteria Clostridium botulinum. This neurotoxin is responsible for botulism. It inhibits the release of the acetylcholine neurotransmitter from axon endings at the neuromuscular junction. There are eight types of BoNT: from A to H. Types A and B are used in headache treatment. The history of the botulinum toxin is long and interesting. The beginning of the history of botulinum toxin began in 1820 when Justinus Kerner published a description of botulism. In 1897, a group of 34 Belgian musicians consumed smoked ham and developed visual and gastrointestinal symptoms characteristic of botulism. The remaining ham was sent to Professor van Ermengem at the University of Ghent. He found the reason for those symptoms, an agent named Bacillus botulinum, though the name was later changed to Clostridium botulinum [8,9]. The clinical use of BoNT began when Dr. Alan Scott used the toxin botulinum in strabismus. Next, the Food and Drug Administration (FDA) approved conducting human research using the toxin for the treatment of strabismus. In 1989, the FDA approved the use of the toxin for the treatment of blepharospasm and hemifacial spasm. One year later, a facial plastic surgeon Dr. Binder observed that some patients who were administered botulinum toxin for cosmetic purposes reported reduced headache frequency. Prospective open-label observational studies confirmed the acceptable safety and tolerability profile of ONA-BoNTA in chronic migraine prophylaxis [10]. In 2010, ONA-BoNTA was reported as effective for the treatment of chronic migraine in the Phase 3 Research Evaluating Migraine Prophylaxis Therapy (PREEMPT) trials [6,7]. Next, it was approved by the European Medicines Agency (EMA) and by the US Food and Drug Administration (FDA) for the prophylaxis of chronic migraines. Its use was endorsed by the National Institute for Health and Care Excellence (NICE) in 2012.

## 3. Tension-Type Headache

Nowadays, ONA-BoNTA is only approved to treat CM. Moreover, it has effects on pain by sensory modulation, therefore, it may also be effective for chronic tension-type headache (CTTH). Tension-type headache (TTH) is another condition with a very high socio-economic consequence. It affects approximately 80% of the general population. The pain in TTH is usually bilateral and is not accompanied by other symptoms [1,11]. TTH affects the temporal and occipital region. CTTH is defined as the occurrence of tension-type headaches at a frequency of ≥15 days per month. Tension-type headache treatment should be multilevel. It often consists of taking pain medication, antidepressants, antiepileptics, acupuncture and attending behavioral therapy. The proposed mechanism of the effect of BoNT in chronic tension-type headaches is the reduction in pericranial muscle tension. It should be emphasized that according to ICHD-3 [1], TTH is divided into two types: those with and those without increased pericranial tenderness upon manual palpation. Not every case of TTH is associated with increased muscle tension, and the name may also suggest “mental and emotional tension”. Pihut et al. [12] suggested that TTH is, in many cases, related to excessive and long-lasting tension within the muscles of the temporomandibular joint. After the injections of botulinum toxin type A, the character of TTH changed, and its intensity decreased. Moreover, a decrease in the daily number of hours and the monthly number of days was also observed [12]. However, there are also studies that do not support the assertion between the use of BoNT and reduction in TTH pain [13,14,15]. To sum up, the currently available scientific data on the efficacy of botulinum toxin in tension-type headaches is ambiguous. BoNT as a potent muscle relaxant may be useful in the treatment of TTH, but these assumptions should be confirmed in large groups of TTH patients. In fact, the pathogenesis of TTH as an idiopathic headache is not related to capillary muscle tone, but the pain is receptor-based and central, although it is not fully understood. More positive results will allow for such a course of action to be recommended. It is worth noting that there are sometimes situations where patients who were initially diagnosed with TTH, after careful history taking, will have the diagnosis adjusted to chronic migraine. BoNT in that situation may be beneficial [16].

## 4. The Use of Botulinum Toxin for the Treatment of Cluster Headache

A cluster headache is a trigeminal autonomic cephalalgia characterized by an extremely painful, strictly unilateral, short-lasting headache attack accompanied by ipsilateral autonomic symptoms. The severity of the disorder has major effects on the patient’s quality of life. The most effective drugs to treat an acute cluster headache attack include 100% oxygen inhalation and triptans sc [17,18]. The efficiency of botulinum toxin in cluster headaches was only observed in some studies [19,20]. Botulinum toxin was administered to the spheno-palatine ganglion and the number of cluster headache attacks was significantly reduced [19]. Another pilot study by Crespi et al. suggested that injection with BoNT to the otic ganglion did not reduce the number of attacks per week 2 months after injection. Perhaps this injection location is not an important target in the treatment of chronic cluster headaches [21]. The injection into the spheno-palatine ganglion is a much more complex procedure than the procedure used for chronic migraine. In practice, botulinum toxin is not used in the treatment of cluster headaches. Further studies are warranted to establish the potential of this possible novel treatment for cluster headaches.

## 5. Botulinum Toxin in the Management of Chronic Migraine

According to the ICHD-3, the diagnosis of chronic migraine was mentioned above. Moreover, the diagnosis of chronic migraine is based on the patient’s history (including a headache diary) and neurological examination. To rule out secondary causes for headaches, magnetic resonance of the brain and lumbar puncture may be necessary. The main goal in the treatment of chronic migraine is to reduce the impact of migraines on patients’ lives [22]. It is very important to keep migraine attacks as rare, short and as least impairing as possible. There are two pillars for the treatment of chronic migraine: treatment of acute attacks and prophylactic treatment. Due to the risk of MOH, prophylactic treatment is important in this group of patients. The overuse of headache medications can be a problem for patients with chronic headache disorders. One of the substances approved by the United States Food and Drug Administration for the treatment of CM, as mentioned before, is ONA-BoNTA [10,23].

Over the years, several studies failed to indicate the positive effects of BoNT on episodic migraines [16,24]. For chronic migraines, the results were inconsistent. All patients responded to the treatment of botulinum toxin type A, but this response was not superior to the placebo [25,26].

A breakthrough in the use of ONA-BoNTA in the treatment of chronic migraine came in 2010. There were two studies: PREEMPT I and PREEMPT II (Phase III Research Evaluating Migraine Prophylaxis Therapy), in which a total of 1384 patients were enrolled [6,7]. In these two studies (PREEMPT I and II), all patients received a minimum intramuscular dose of 155 units of ONA-BoNTA administered to 31 injection sites across seven head and neck muscles using a fixed side. The minimum dose was 155 units, and the maximum dose was 195 units. The main results from the PREEMPT clinical program have established that ONA-BoNTA is a safe, well-tolerated and effective headache prophylactic treatment for CM [6,7,27]. PREEMPT results support previous studies, which identified chronic migraine patients as most likely to benefit from ONA-BoNTA treatment [25,26,28]. ONA-BoNTA dosing for CM by muscle using the PRREMPT injection program is shown in Table 1. When deciding on the dose and location of additional onabotulinumtoxin type A, the location of the patient’s predominant pain and the severity is important.

**Figure 1:**
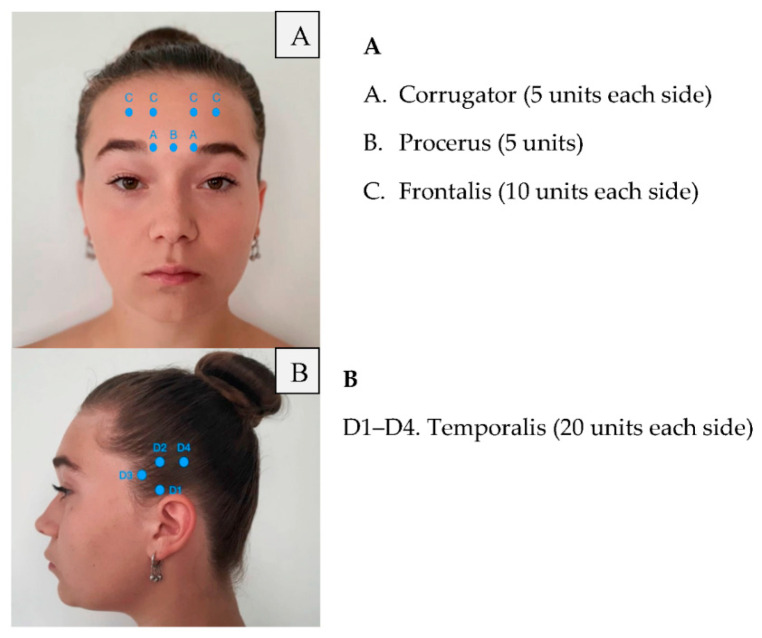
Frontalis and temporalis injections of botulinum toxin.

**Figure 2:**
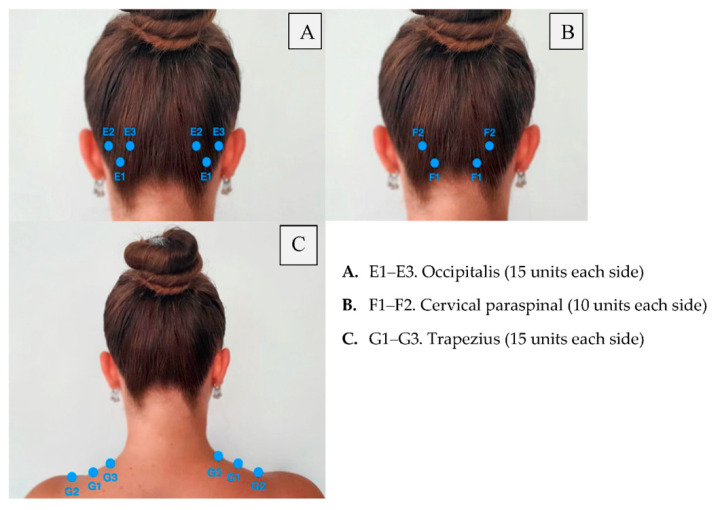
Posterior injections of botulinum toxin.

**Figure 3:**
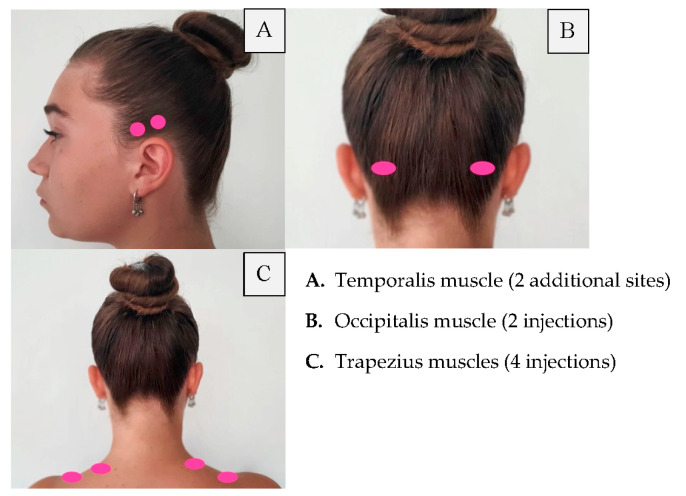
Follow the pain injection sites.

Retreatment with botulinum toxin occurs at 12-week intervals. Meanwhile, patients should keep a headache diary. It is recommended to repeat the injection every 12 months, at least three times. Moreover, the patient should receive additional injections in areas where, in particular, they have pain. This additional treatment strategy is called “Follow the pain”, and it was also used by many of the PRREMPT testing sites before FDA approval. Injection points of the botulinum toxin are presented in Figure 1, Figure 2 and Figure 3, where a patient with chronic migraine who was treated with botulinum toxin with a positive therapeutic effect is shown.

## 6. Mode of Action of ONA-BoNTA

When onabotulinumtoxin type A is injected into the extracellular space, the heavy chain of the toxin binds to receptors on the C-fiber nerve terminal. Then, ONA-BoNTA is endocytosed and enters the nerve terminal enclosed in a vesicle. Next, botulinum toxin uses its endopeptidase light chain to deactivate the synaptosomal protein known as SNAP-25. This protein is located on the cell membrane, and its deactivation prevents the release of acetylcholine. Moreover, CGRP calcitonin gene-related peptide and other neuropeptides cannot be released [8,23,29,30]. The ability of ONA-BoNTA to block CGRP is likely critical to its therapeutic role in migraines. More recent studies show that botulinum toxin modifies the release of neurotransmitters that are relevant in the transduction of pain (such as CGRP, substance P and glutamate) [8,30,31]. ONA-BoNTA reduces the number of pain signals that reach the brain and consequently prevents the activation and sensitization of central neurons [30].

## 7. Conclusions

This article presented the primary headaches, otherwise named idiopathic, which are a large group of diseases. The etiology is unclear, and the headache is not a symptom of another known disease. The most common of these are migraine, tension-type headache and cluster headache. Many of these patients seek dental care because orofacial pain is a common presenting symptom. It would be advisable for a specialist in orofacial pain to know the criteria for the diagnosis of headaches and for headache physicians to know the semiologic aspects of orofacial pain. It is, therefore, necessary to differentiate these symptoms. Moreover, botulinum toxin would be helpful in treating bruxism or other occlusal disorders. This problem may be covered in other articles and would be helpful, especially in dental practice.

It has been well established that botulinum toxin may be an effective prophylactic treatment for chronic headache types. Botulinum toxin serotype A was originally used in neurology for the treatment of dystonia and blepharospasm. It is also widely used in the treatment of the disease of the rumen and temples of the temporomandibular muscles, as presented by Ferrillo M [32].

Currently, it is clinically used as an attractive alternative for patients with chronic migraine who do not respond positively to other drugs. ONA-BoNTA is effective in CM, not only in headache frequency and pain intensity but in other parameters, including quality of life. Recent and future developments in other headache disorders are still discussed. However, the results of treatment are dependent on the dose, location of injection and the number of cycles.

## Figures and Tables

**Table 1:** OnabotulinumtoxinA dosing in chronic migraine according to protocol PRREMPT [24].

Area of Injection	Recommended Dose
Frontalis Corrugator Procerus Occipitalis Temporalis Trapezius Cervical paraspinal muscle group	20 units (4 sites) 10 units (2 sites) 5 units (1 site) 30 units (6 sites) + 10 units in 2 sites (follow the pain areas—optional injections) 40 units (8 sites) + 10 units in 2 sites (follow the pain areas—optional injections) 30 units (6 sites) + 20 units in 4 sites (follow the pain areas—optional injections) 20 units (4 sites) **Summary: 155–195 units**

## Data Availability

Not applicable.
